# A Pelican Tarsometatarsus (Aves: Pelecanidae) from the Latest Pliocene Siwaliks of India

**DOI:** 10.1371/journal.pone.0111210

**Published:** 2014-11-03

**Authors:** Thomas A. Stidham, Kewal Krishan, Bahadur Singh, Abhik Ghosh, Rajeev Patnaik

**Affiliations:** 1 Key Laboratory of Vertebrate Evolution and Systematics, Institute of Vertebrate Paleontology and Paleoanthropology, Chinese Academy of Sciences, Beijing, China; 2 Department of Anthropology, Panjab University, Chandigarh, India; 3 Department of Geology, Panjab University, Chandigarh, India; Bournemouth University, United Kingdom

## Abstract

We report a new fossil specimen of a pelican from the Tatrot Formation of the Siwalik Hills, India. It likely represents *Pelecanus sivalensis* Davies, 1880, the smaller of the two previously published species from the Siwalik Group stratigraphic sequence. This complete tarsometatarsus is the first fossil bone of a pelican collected in India for over 100 years. It is from the latest Pliocene (∼2.6 Ma), and is the youngest pelican fossil from the region. The new specimen exhibits a derived distoplantar ‘slant’ to the plantar margin of the medial crest of the hypotarsus, and a combination of features related to the morphology of the hypotarsus, the distal foramen, trochleae, and overall size that allow further differentiation from known tarsometatarsi of fossil and extant pelicans, including the three species of extant pelicans that occur in India (*Pelecanus crispus*, *P*. *onocrotalus*, and *P*. *philippensis*). It is of appropriate size for *Pelecanus sivalensis*, which to date has been known only by fragments of other skeletal elements of the wing, leg, and shoulder girdle. Thus, the observation that this tarsometatarsus is morphologically distinct from those of known pelicans provides further support for the distinctiveness of at least one extinct species of pelican from the Siwalik Group sediments. While the morphology of the tarsometatarsus allows for separation from other taxa known from tarsometatarsi, we found no clear shared derived states to place this taxon with any confidence in a phylogenetic context relative to any other pelican species, or even determine if it is part of the crown group of Pelecanidae. However, published molecular data are consistent with an origin of the crown clade prior to the Pleistocene, suggesting (along with one morphological character) the possibility that this species belongs to the Old World clade of pelican species.

## Introduction

Pelicans are large birds that inhabit fresh water, brackish, and marine habitats on all continents except for Antarctica. They have webs linking all toes and distinctive pouches on their jaw. Extant pelicans comprise eight recognized species that are supported by molecular and morphological data [1], [2]. Those species include the extant Old World species, the Dalmatian Pelican (*Pelecanus crispus*), Spot-billed Pelican (*P*. *philippensis*), Pink-backed Pelican (*P*. *rufescens*), Australian Pelican (*P*. *conspicillatus*), and Great White Pelican (*P*. *onocrotalus*), and also include the New World species, the Brown Pelican (*P*. *occidentalis*), Peruvian Pelican (*P*. *thagus*), and American White Pelican (*P*. *erythrorhynchos*). The recent molecular phylogeny of extant pelicans [2] differs from previous phylogenetic hypotheses and supports the presence of a main division (basal split) among pelican species composed of New World species and Old World species, respectively. While those authors did not specifically discuss the timing of the origin of the crown group of Pelecanidae (i.e. the most recent common ancestor of the eight extant species), the genetic distances they presented separating the extant species are consistent with an origin of the clade before the Pleistocene.

The pelican fossil record is not particularly rich, although extinct taxa have been described from rocks as old as the Oligocene [3]–[6]. The oldest fossil pelican exhibits the distinctive skull shape present in all extant pelicans indicating its early origin (and likely presence in all known fossil pelican taxa) [3]. While the fossil record of pelicans extends into the Oligocene [3], the Neogene and Quaternary records of fossil pelicans are not very plentiful. However, fossil Neogene and Quaternary pelicans are known from all continents except Antarctica. Most fossil species have been placed in *Pelecanus*, although the genus *Miopelecanus* was erected to accommodate a few species [7] (but see ref. [3] for more discussion). European fossil pelicans are known from the Miocene (*Pelecanus intermedius*, *P*. *gracilis*, and *P*. *fraasi*) and Pliocene (*P*. *odessanus*) [5]. Australia and New Zealand species include fossils from the Oligo-Miocene (*P*. *tirarensis*) and the Quaternary *P*. *cadimurka* and *P*. *novaezeelandiae* [5]. All of the known Neogene fossils of pelicans in Asia derive from the Siwalik Hills in India and Pakistan and comprise material allocated to *P*. *cautleyi* and *P*. *sivalensis* [5]. Pleistocene fossils from Africa (Olduvai) were named *P*. *aethiopicus*, but that material may be synonymous with the extant *P*. *onocrotalus* [8]. The pelican record from North America is limited to the Pliocene *P*. *halieus* and *P*. *schreiberi* [5], [6]. The South American record is limited and includes the Miocene holotype of *Liptornis hesternus* (a cervical vertebra) that may not even be from a pelican [5]. Fossils that have been identified as extant species also are known from the late Neogene and Quaternary (e.g., *Pelecanus onocrotalus* ref. [8]).

Fossil pelicans are rare in Asia, and currently are documented only from several fossils from the Siwalik Hills in Pakistan and India [1], [9], [10]. Davies [9] named two species, *Pelecanus cautleyi* Davies, 1880 and *Pelecanus sivalensis* Davies, 1880, based on distal ulnae from the Siwalik Hills area, and he reported them as being early (lower) Pliocene in age (reiterated by Lydekker [10]). Lydekker [10] referred additional material to *Pelecanus cautleyi* including fragments of radii and femora, and a fragment of a radius to *Pelecanus sivalensis* based on its relatively smaller size. Harrison and Walker [1] referred further fragments of a coracoid, humerus, and ulna to *Pelecanus sivalensis* from the late Miocene of Pakistan [11]. The morphological data available in those specimens is limited and discussion of their affinities has mostly focused on their size. Both of these extinct species are smaller than that of the extant southern Asian *Pelecanus onocrotalus* (*P*. *mitratus* in their usage; [9], [10]). Harrison and Walker [1] stated that *Pelecanus cautleyi* is similar in size to the extant *Pelecanus conspicillatus* and that *Pelecanus sivalensis* is similar in size to *P*. *occidentalis*. Olson [5] doubted that both species were biologically distinct suggesting that only one pelican species was likely to be represented (not two).

**Figure 1:**
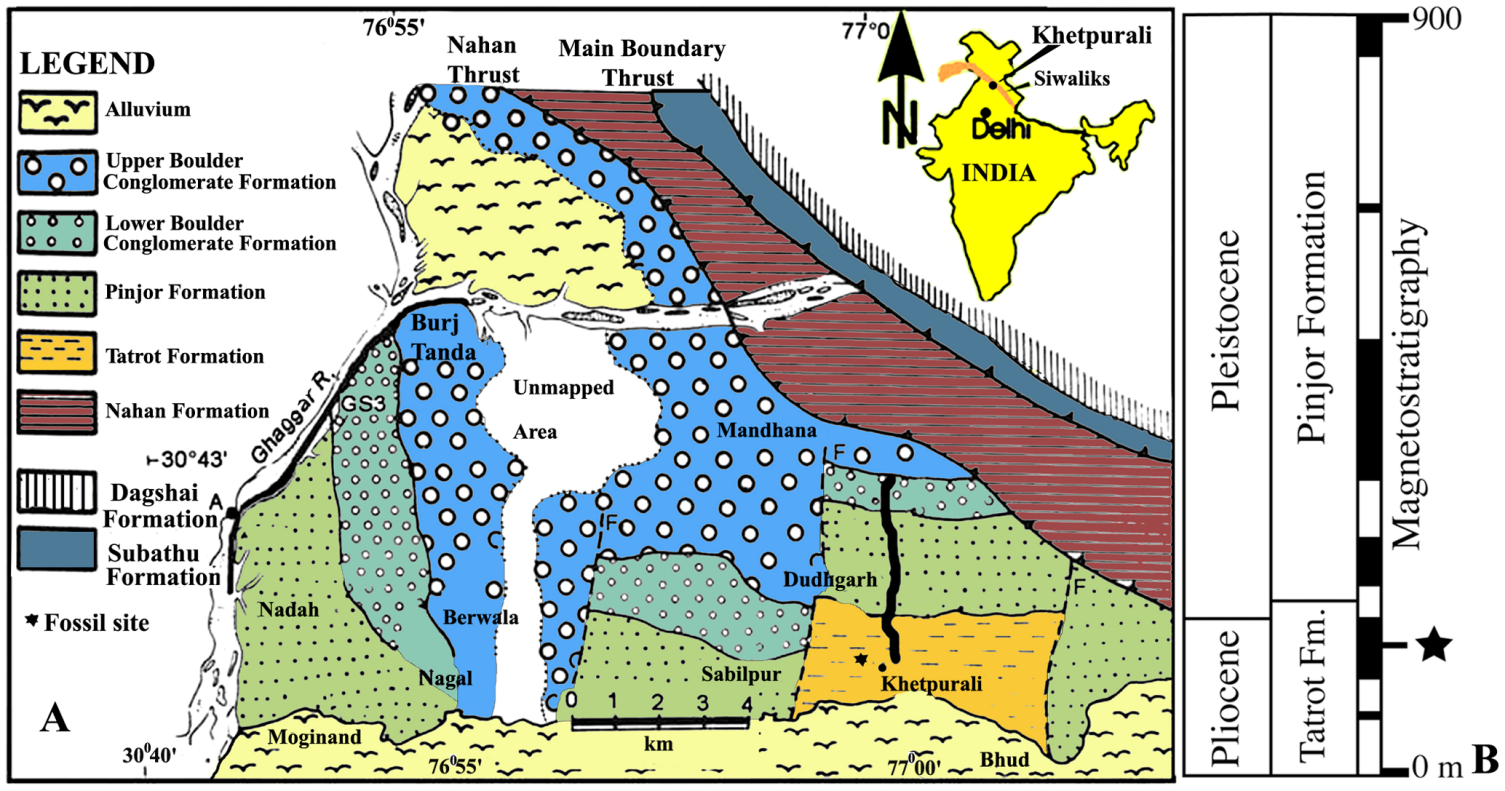
Geology, stratigraphy and magnetostratigraphy of the Khetpurali section, India, showing the fossil site and its approximate age. The geological map is modified from Kumar and Tandon [25]. B. The Khetpurali section, its magnetostratigraphy, and its correlation with the GPTS (data from Tandon et al. [16] and Gradstein et al. [20]).

Most of the pelican material from the Siwalik Hills derives from the collections made by Sir Proby Thomas Cautley [10] in colonial India (including what is today India, Pakistan, and Bangladesh). Some of the fossils collected during this time period (by a variety of people) are from within the boundaries of modern India, and some are from within what is today Pakistan (see below). Even though the Indian pelican fossils all derive from the Cautley collection, both he and Falconer did not keep detailed stratigraphic data on the specimens in their collections [12]. Therefore, determining the exact age of the holotypes and referred material of the pelicans from the Indian Siwalik Hills is not possible with any certainty. Since the 19th century, the geology and stratigraphy of the Siwalik Hills has been studied extensively and revised (see ref. [12] for a partial review), and it is now known that the stratigraphic sequence includes both Miocene and Pliocene sediments. Without better stratigraphic data on the fossil pelican bones among the Cautley material, it is not clear whether the specimens are Pliocene or late Miocene in age. However, some authors (e.g., [5]) have continued to cite the holotypes and referred material of the Siwalik species as of lower Pliocene age (i.e. the age assigned in 1880), while Harrison and Walker [1] suggested that the specimens were younger at late Pliocene or early Pleistocene (consistent with the current age estimate of the Tatrot Formation, Figure 1). However, Harrison and Walker [1] presented no justification for that age.

Some of the non-pelican avian material from the Siwalik Hills, including a specimen of a *Mergus* sp., is from Asnot (Hasnot) part of the Punjab region, in what is now Pakistan [13], and derives stratigraphically from the Dhok Pathan Formation, which in Pakistan is proposed to extend between 9.8 and 3.3 Ma (Late Miocene to early Late Pliocene) [14]. However, in India, the Dhok Pathan Formation is thought to range between 10 and 5.5 Ma in age (i.e. solely Miocene in age) [15]. The more recently collected Miocene avian material in Pakistan [1] is even older [11]. Despite those ambiguities, it seems clear that the tarsometatarsus from the Khetpurali section described below is younger than the Dhok Pathan specimens, and supports the presence of a distinct extinct pelican species (either *Pelecanus cautleyi* or more likely *P. sivalensis*).

Geological context and faunal association of the pelican tarsometatarsus: The Siwalik Group comprises freshwater deposits over 6000 m thick that are exposed along the Himalayan foothills and range in age from ∼18 to 0.5 Ma (see ref. [15] for a review). The Siwalik Group is subdivided into the Lower, Middle, and Upper Subgroups based on faunal assemblages and sedimentary facies. The Upper Siwalik Subgroup is divided into Tatrot (∼5.26 to ∼2.58 Ma), Pinjor (∼2.58 to ∼1.7 Ma), and Boulder Conglomerate Formations (∼1.7 to ∼0.5 Ma) (see ref. [15] for a review). The Upper Siwalik Subgroup, as exposed around Khetpurali (Figure 1), includes outcrop of the Tatrot Formation (210 m thick) that forms an anticline, and is characterized by the presence of fine, medium, and coarse-grained, grey sandstones and variegated mudstones deposited mainly in low sinuosity streams [16], [17]. This section was paleomagnetically dated by Tandon and coauthors [16]. The Tatrot fauna of this area comprises *Stegodon bombifrons, Pentalophodon khetpuralensis*, *Hipparion antelopinum, Hipparion theobaldi, Hexaprotodon sivalensis*, and *Camelus sivalensis* [18], [19]. The upper contact with the Pinjor Formation is transitional from grey beds that gradually disappear to dominant brown sandstone/mudstone. In the Khetpurali Section, the Pinjor Formation is about 680 m thick and consists of brown to greyish-brown, fine, medium, and coarse-grained sandstones, multistory sandstones, pebbly sandstone, and pedogenic and non-pedogenic overbank facies deposited in high gradient, low sinuosity streams, mainly of piedmont drainage [16], [17]. The mammalian taxa known from the Khetpurali Region include *Stegodon insignis, Archidiskodon planifrons, Elephas hysudricus, Equus sivalensis, Leptobos*, and *Bos* [16], [19]. The overlying (∼155 m thick) Boulder Conglomerate Formation comprises pebbles, cobbles, and boulders embedded in sandy to silty matrix with interstratified sandstone and clay beds, is largely unfossiliferous, and was deposited mainly in braided river channels and proximal alluvial fans. The pelican fossil described here derives from a bone-bearing horizon in the Tatrot Formation (Figure 1B) in the Khetpurali section (∼2.6 Ma) that has yielded an associated fauna that includes crocodile, crab, and fish remains, suggesting a depositional setting of a floodplain pond. This horizon has a magnetic polarity age of the last normal polarity magnetochron within the Pliocene (using ref. [20] stratigraphic terminology and chronology), and thus the fossil is from near the very end of the Pliocene.

Institutional abbreviations: IVPP—Institute of Vertebrate Paleontology and Paleoanthropology, Chinese Academy of Sciences, Beijing; MVZ—Museum of Vertebrate Zoology, University of California, Berkeley, USA; UCMP—University of California Museum of Paleontology, Berkeley, USA; USNM—United States National Museum, Washington, D.C., USA. Access to the MVZ and UCMP collections were provided by Carla Cicero and Patricia Holroyd (respectively). No permits were required for the described study, which complied with all relevant regulations. Osteological terminology follows Baumel and Witmer [21] with English equivalents of the Latin terms.

## Systematic Paleontology

Aves Linnaeus 1758

Pelecaniformes Sharpe 1891

Pelecanidae Rafinesque 1915

*Pelecanus sivalensis*? Davies 1880

Referred Specimen: Specimen No. KP/KK/BS/100 (KP-Khetpurali, KK-Kewal Krishan, BS-Bahadur Singh) is a left tarsometatarsus and is stored in the Museum of the Department of Anthropology at Panjab University, Chandigarh, India.

**Figure 2:**
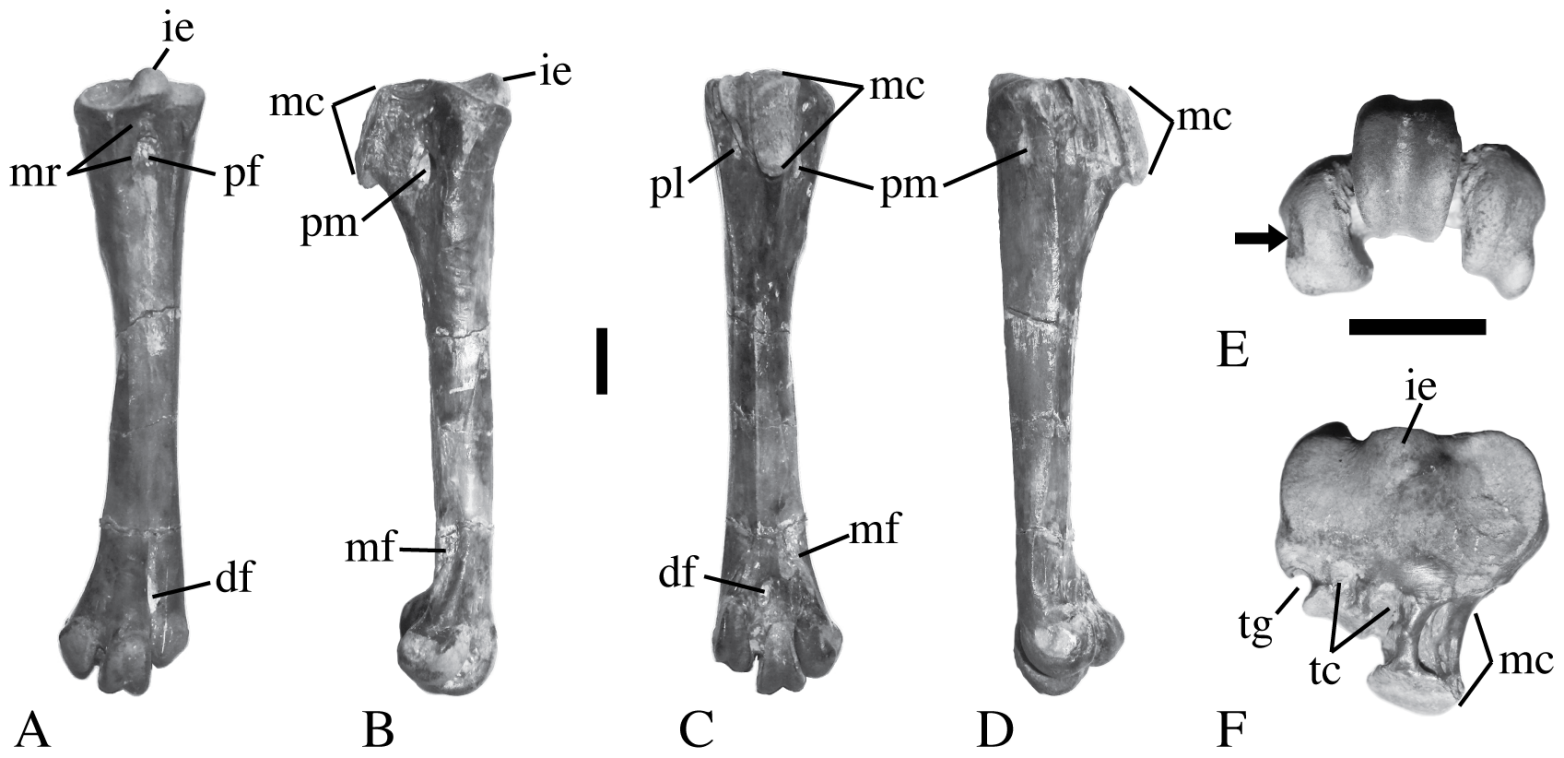
Tarsometatarsus KP/KK/BS/100 tentatively referred to *Pelecanus sivalensis*. A. dorsal; B. medial; C. plantar; D. lateral; E. distal; and F. proximal views. The arrow in E indicates the concave notch in the medial side of trochlea II that is a Steganopodes (sensu Smith [22]) synapomorphy. The scale bar is 1 cm (with one scale bar for parts A–D and one for E–F). Abbreviations: df—distal foramen; f—small pneumatic foramen; ie—intercondylar eminence; mc—medial crest of the hypotarsus; mf—fossa for metatarsal I; mr—ridge medial to the dorsal pneumatic foramen that is part of the extensor retinaculum attachment; pf—dorsal pneumatic foramen where the proximal foramina would be in other taxa; pl—lateral plantar opening of the proximal foramen; pm—medial plantar opening of the proximal foramen; r—ridge on the medial hypotarsal crest that bounds a concave area to its medial side; tc—tendinal canal opening; tg—tendinal groove.

Taxon diagnosis of the tarsometatarsus: The tarsometatarsus exhibits the extension of trochlea II distal to the level of trochlea IV that is a derived feature among Pelecaniformes (Smith's [22] Steganopodes) (and some traditional ciconiiform birds), and the concave medial distal face of trochlea II (i.e. a notch) that is a synapomorphy of what Smith [22] considered Steganopodes (Figure 2, [22]). That latter character is also present convergently among Anseriformes. The area on the dorsal proximal surface where the proximal foramina would be (in other pelecaniform taxa) is recessed into a single pneumatic opening, as in all extant species of *Pelecanus*. That character is unique to species of *Pelecanus* among traditional Pelecaniformes (and Smith's [22] extant Steganopodes), and so it is a synapomorphy of all or part of Pelecanidae).

The specimen has a nearly unique character among known pelicans in that the plantar edge of the enlarged medial hypotarsal crest is not parallel with the shaft of the bone, but instead slopes distoplantarly with the distal end plantar to the proximal end (Figure 2). That trait also is present in *Pelecanus occidentalis urinator* (MVZ 140909), but is absent in all (other) extant species (including specimens of *P*. *occidentalis californicus*) and in the known tarsometatarsi of fossil pelicans (e.g., [23]). Comparisons among the tarsometatarsi of extant and extinct species of pelicans indicate that this bone can be further separated from those taxa with a combination of characters, including size (see below and Tables 1 and 2). If this tarsometatarsus is from one of the two previously named species from the Siwalik Group, then these characters suggest that at least one of them is a biologically distinct species from other described species (extinct and extant).

**Table 1:** Tarsometatarsus measurements (in mm) of some extant and extinct pelican species.

Species	Specimen No.	Length	Distal Width	Trochlea III Width
*P*. *sivalensis*?	KP/KK/BS/100	94.6 L	19.9	7.6
*P*. cf. *cadimurka*	UCMP 60578		18.8	7.2
*P*. cf. *cadimurka*	UCMP 60577		18.5	7.5
*P*. *crispus*	USNM 557493	131.9 L		
		131.9 R		
*P*. cf. *conspicillatus*	UCMP 56322			10.3
*P*. *conspicillatus*	MVZ 143249	132.0 L	24.5	9.8
		132.6 R	24.4	9.9
*P*. *conspicillatus*	MVZ 143248	122.6 L	21.6	8.4
		123.0 R	21.4	8.3
*P*. *conspicillatus*	MVZ 143245	117.4 L	21.1	8.4
		117.8 R	21.1	8.1
*P*. *conspicillatus*	USNM 554904	114.9 L		
		115.8 R		
*P*. *conspicillatus*	USNM 554118	115.3 L		
		115.5 R		
*P*. *erythrorhynchos*	MVZ 180137	108.4 L	21.5	8.6
		109.6 R	21.1	8.2
*P*. *erythrorhynchos*	MVZ 182793	124.2 L	24.1	9.0
		124.7 R	24.1	8.8
*P*. *occidentalis californicus*	MVZ 66500	91.2 L	20.7	7.3
		91.5 R	20.5	7.3
*P*. *occidentalis californicus*	MVZ 125367	77.4 L	19.2	6.8
		77.5 R	19.2	6.8
*P*. *occidentalis urinator*	MVZ 140909	78.3 L	20.3	7.1
*P. onocrotalus*	USNM 17334	148.6 L		
		148.3 R		
*P. onocrotalus*	USNM 558366	122.1 L		
		122.5 R		
*P*. *philippensis*	IVPP 1031	109.7 L	23.1	9.4
		109.6 R	23.1	9.3
*P*. *rufescens*	USNM 18737	86.1 L		
		86.2 R		
*P*. *rufescens*	USNM 18735	85.6 L		
		85.6 R		
*P*. *tirarensis*	UCMP 57014		19.6	7.5

**Table 2:** Variable characters of the pelican tarsometatarsus and their distribution across *Pelecanus* species.

Species	1	2	3	4	5
*P*. *sivalensis*?	1	0	1	0	0
*P*. cf. *cadimurka*	?	?	1	0	0
*P*. cf. *conspicillatus*	?	?	1	0	?
*P*. *conspicillatus*	0	0&1	1	1	0
*P. crispus*	0	-	-	-	0
*P*. *erythrorhynchos*	0	0	0	0	1
*P*. *occidentalis californicus*	0	0&1	1	0	1
*P*. *occidentalis urinator*	1	0	1	0	1
*P. onocrotalus*	0	-	-	-	0
*P*. *philippensis*	0	1	0	0	0
*P. rufescens*	0	-	-	-	0
*P*. *tirarensis*	?	?	1	1	?

See text and Table 1 for the full list of specimens examined. The states in *P. crispus*, *P*. *onocrotalus*, and *P*. *rufescens* are scored from illustrations in ref. [23].

Characters:

1. Medial hypotarsal crest: parallel to the tarsometatarsus shaft (0); ‘slanting’ with the proximal end located more dorsal relative to the distal end (1).

2. Attachment of the extensor retinaculum just medial to the dorsal pneumatic opening on the proximal end of the tarsometatarsus: short, either not reaching or just reaching distally to the proximodistal midpoint of the pneumatic opening (0); longer, extending distal to the proximodistal midpoint of the opening (1).

3. Trochlea II and IV: extend about equally far distally (0); trochlea II extends more distal than trochlea IV (1).

4. Concave area on the medial face of trochlea II plantar to the collateral ligamental pit: absent (0); present (1).

5. Proximal face of the medial hypotarsal crest: concave with a ridge bounding its edge (0); not concave and ridge absent (1).

Harrison and Walker [1] stated that the holotype ulna of *Pelecanus cautleyi* is similar in size to the corresponding element in *Pelecanus conspicillatus*, and that of *Pelecanus sivalensis* is more similar in size to the smaller *Pelecanus occidentalis* from North America. The size of the new fossil tarsometatarsus is closer to the range found in *Pelecanus occidentalis* than to that of the larger *Pelecanus conspicillatus* (Table 1). Given those size relationships (and assuming allometry among the species), this tarsometatarsus likely belongs to the smaller Siwalik species, *Pelecanus sivalensis*. This identification is tentative because of the lack of corresponding skeletal elements between this tarsometatarsus and the holotype (and referred material) for *P. sivalensis*.

**Figure 3:**
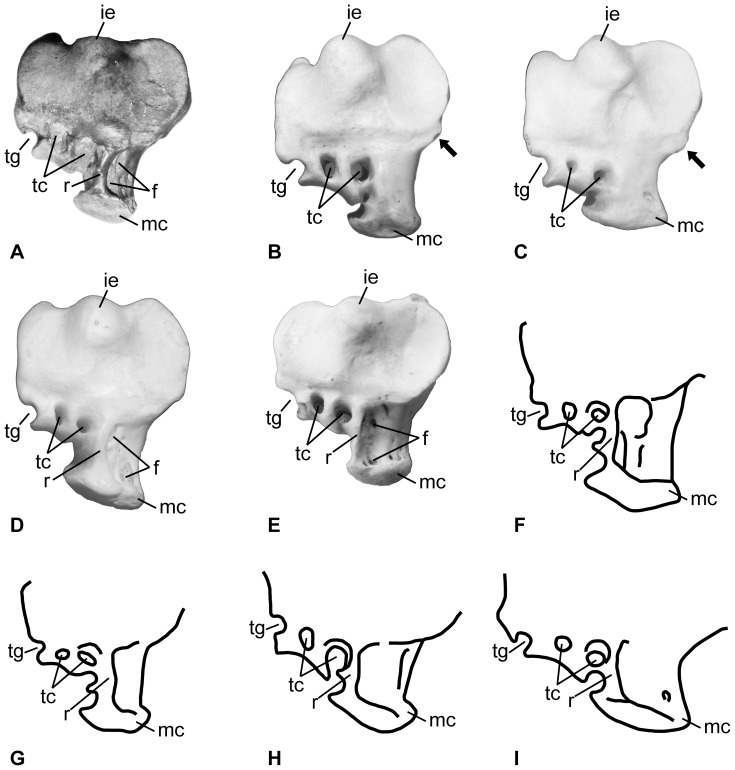
Comparison of the proximal end of the tarsometatarsus of various pelican species. A. *Pelecanus sivalensis*? (KP/KK/BS/100); B. *Pelecanus occidentalis californicus* (MVZ 66500); C. *Pelecanus erythrorhynchos* (MVZ 182793); D. *Pelecanus conspicillatus* (MVZ 143248); E. *Pelecanus philippensis* (IVPP 1031); F. *Pelecanus crispus*; G. *Pelecanus rufescens*; H. *Pelecanus aethiopicus*; I. *Pelecanus onocrotalus*. Images in F–I are redrawn from Harrison and Walker [23]. The arrow in B and C indicates the plantar medial extension of the proximal end discussed in the text. The individual photographs and drawings are set to be roughly equal in width to enhance morphological differences (rather than those of size). See Figure 2 for explanation of the abbreviations.

Description: There is one large subcircular pneumatic opening on the dorsal surface where the proximal foramina would pass through the bone in other avian taxa. The short inner ridge (the attachment of the extensor retinaculum medial to the pneumatic opening) extends distally to approximately the level of the proximodistal midpoint of that opening. The plantar opening of the distal foramen is ovoid in outline. Trochlea II extends further distally than trochlea IV, but not as far distally as trochlea III. The plantar edge of the medial hypotarsal crest is thickened and is not parallel with the shaft of the bone, but instead exhibits a ‘slant’ with the proximal end located more dorsal relative to the more plantar distal end (Figure 2). In plantar view, the medial plantar edge of the medial hypotarsal crest is concave and its lateral edge is convex. The proximal face of the medial hypotarsal ridge has a concave semicircular area, is enclosed laterally by a low ridge, and has pneumatic foramina bordering the concave area adjacent to the ridge (Figure 3). The hypotarsus has two fully enclosed canals and a lateral groove. The medial enclosed canal is caudal to the intertrochlear eminence, and the second canal is lateral to that position, but is medial to a tendinal groove at the lateral plantar corner of the bone. The hypotarsus plantar to the lateral canal is concave and supported other tendons. There is a pair of tendinal grooves on the lateral surface of the medial hypotarsal ridge that is plantar to the medial enclosed canal and they are oriented with one plantar to the other.

Comparisons: This fossil cannot be compared directly to the named pelican species from the Siwalik Group (*Pelecanus sivalensis* Davies 1880 and *Pelecanus cautleyi* Davies 1880) because those species are known from material that does not include the tarsometatarsus. Their size is given as smaller than the extant *Pelecanus onocrotalus* by Louchart [8], Davies [9], and Lydekker [10]. Harrison and Walker [1] stated that the ulnae of *Pelecanus sivalensis* and *P. cautleyi* are most similar in size to the corresponding elements in *Pelecanus occidentalis* and *P. conspicillatus*, respectively. However, Rich and Van Tets [4] cite Miller [24] as stating that both species from the Siwalik Group are smaller than *Pelecanus conspicillatus*, but support for that statement does not appear to be in that publication (except via the tables of measurements), only a comparison to *Pelecanus roseus* (i.e. *Pelecanus onocrotalus roseus*).

Olson [6] stated that the fossil taxon *Pelecanus schreiberi* is larger than the extant Asian pelican species (*P. melanogaster* and *P. philippensis*), and thus would be larger than the species represented by this Siwalik's tarsometatarsus. The tarsometatarsus of the Oligocene Australian fossil *Pelecanus tirarensis*, (UCMP 57014 cast of the holotype) is roughly the same size as the tarsometatarsus from the Siwalik Group described here, but the medial face plantar to the ligament pit on trochlea II is concave unlike in the Siwalik Group specimen (but a similar state is in *Pelecanus conspicillatus*). That concave area was illustrated by Miller (fig. 1A in [24]). Also, the furrow on trochlea III in *Pelecanus tirarensis* is much more deeply incised on its dorsal side and distal end, and the intertrochlear incisures are relatively wider than the states in KP/KK/BS/100. A second Australian fossil species, the Plio-Pleistocene *Pelecanus cadimurka*, has tarsometatarsi (e.g., UCMP 60578) roughly the same size as the tarsometatarsus from the Siwalik Group, but only the trochleae are preserved (in the type of this species). Its proximal end of the plantar side of trochlea III is squared off (rather than the rounded state in the Indian specimen). The plantar opening of the distal foramen is closer to trochlea III than in KP/KK/BS/100, but the furrow on trochlea II extends more dorsal in UCMP 60578. Additionally, UCMP specimen 60577 of *Pelecanus* cf. *cadimurka* has a relatively mediolaterally narrower trochlea IV and a wider medial intertrochlear incisure than the Indian tarsometatarsus.

Harrison and Walker [1] stated that the tarsometatarsus of *Pelecanus odessanus* from the Miocene of the Ukraine is longer than any extant species, and thus would be much longer than this Siwalik Group fossil (see Table 1 for length comparisons to extant species).

The extant Australian Pelican *Pelecanus conspicillatus* (MVZ 143245, 143248, and 143249) has tarsometatarsi much larger than the fossil (Table 1). The inner ridge of the extensor retinaculum forms the medial edge of the pneumatic foramen in the extensor sulcus (rather than being medial to the foramen). That ridge also extends distally past the proximodistal midpoint of the pneumatic opening (also in MVZ 143245, but is shorter, not reaching the midpoint in MVZ 143248), and the ridge's length is clearly variable intra- and interspecifically (see below). The furrow on trochlea III is wider and deeper on the dorsal and distal sides than it is in the tarsometatarsus from the Siwalik Group. The plantar opening of the distal foramen is an elongate slit (unlike the fossil). The medial side of trochlea II has a medial plantar concavity similar to that of the extinct *Pelecanus tirarensis* (see below, and illustrated in fig. 1 in [24]), but it is less distinct in the female specimens examined (MVZ 143248 and 143245). The medial hypotarsal crest is parallel to the shaft of the tarsometatarsus. The plantar opening of the medial proximal foramen is very large and located more proximal than it does in the Indian fossil. The foramen is a bit smaller in the females examined (MVZ 143248 and 143245) than it is in the male examined. The proximal face of the medial hypotarsal crest has pneumatic foramina, but the ridge surrounding the concave proximal face of that crest is more squared off than the rounder condition in the fossil.

The fossil specimen UCMP 56322 initially was referred to *Pelecanus grandiceps* by Miller [24], but Rich and Van Tets [4] placed it in the extant species *Pelecanus conspicillatus*. This UCMP specimen is much larger than the Siwalik tarsometatarsus, and it exhibits a trochlea II that extends distally almost equal to that of trochlea III. UCMP 56322 lacks the plantar concave area on trochlea II that is present in the older *Pelecanus tirarensis* (also unlike the extant specimens of *Pelecanus conspicillatus* examined above). That character difference may suggest that this specimen does not belong to *Pelecanus conspicillatus*, as originally suggested by Miller [24], but a larger sample size is needed to verify that speculation.

The extant American White Pelican *Pelecanus erythrorhynchos* (MVZ 180137 and 182793) has tarsometatarsi much bigger than the present fossil. The plantar opening of the distal foramen is proximodistally elongate, but not slit-like. The lateral intertrochlear incisure is relatively wider than the state in the fossil. The inner ridge of the extensor retinaculum is short and does not reach the proximodistal midpoint of the pneumatic foramen. Trochlea II and IV have equal distal extension. The plantar margin of the medial hypotarsal crest is parallel to the shaft, and is relatively longer than the same structure in the fossil. The plantar opening of the medial proximal foramen is closer to the proximodistal midpoint of the hypotarsus than the more distal position in the fossil tarsometatarsus. The foramen is larger in the male specimen (MVZ 182793) than in the female (as was also observed in *Pelecanus conspicillatus*). The proximal face of the medial hypotarsal crest lacks the raised ridge surrounding a fossa. In proximal view, the bone has a plantar medial squared off projection that is absent in the fossil (Figure 3).

The extant Brown Pelican *Pelecanus occidentalis californicus* (MVZ 66500 and 125367) has tarsometatarsi approximately the same distal width, but slightly shorter than the fossil (Table 1). The dorsal proximal pneumatic opening for the proximal foramina is proximodistally elongate, and it has a distal extension on the medial side (absent in MVZ 125367). The length of the inner medial ridge of the extensor retinaculum varies with it longer than the state in the fossil in MVZ 66500 and shorter in MVZ 125367 (not reaching the proximodistal midpoint of the opening). The plantar opening of the distal foramen is rounded. The medial side of trochlea II lacks the plantar concave area present in *Pelecanus tirarensis*. The proximal face of the medial hypotarsal crest lacks the concave area, adjacent ridge, and foramina present in the fossil. There is a smaller plantar medial squared off projection (in proximal view) than that in *Pelecanus erythrorhynchos* (see above and Figure 3). The plantar opening of the medial proximal foramen is closer in size to the state in the fossil. The medial hypotarsal crest is parallel to the tarsometatarsal shaft. There are pneumatic foramina near proximal margin of the dorsal pneumatic opening for the proximal foramina (that are absent in the Indian fossil). There is a small pneumatic foramen proximal to the plantar opening of the medial proximal foramen. The plantar opening of the medial proximal foramen is large in MVZ 125367, and it extends distal to the medial hypotarsal crest (unlike the Siwalik specimen).

The extant *Pelecanus occidentalis urinator* from the Galapagos Islands (MVZ 140909) has tarsometatarsi shorter than the fossil. There is a deep concave groove distal to the proximal dorsal pneumatic opening for the proximal foramina. There are no pneumatic foramina proximal to the dorsal pneumatic opening as in *Pelecanus occidentalis californicus*. The inner ridge of the extensor retinaculum is short. The medial hypotarsal crest is slanted distoplantarly like the state in the fossil and unlike *Pelecanus occidentalis californicus*. The plantar openings of the proximal foramina are distal to the medial hypotarsal crest, and the crest is rounder, less elongate than in the fossil and other extant specimens examined. The plantar distal foramen is rounded in outline. The proximal face of the medial hypotarsal crest is flattened and lacks the ridge bounding its edge. This specimen shares the small foramen proximal to the plantar opening of the medial proximal foramen as in the other subspecies.

The extant Spot-Billed Pelican *Pelecanus philippensis* (IVPP 1031) has tarsometatarsi larger than the fossil, and trochlea II extends about as far distally as trochlea IV. The proximal pneumatic opening is more proximodistally elongate than the more subcircular outline present in the fossil. The short inner ridge of the extensor retinaculum is longer, extending distal to the proximodistal midpoint of the pneumatic opening. The intertrochlear incisures are wider than in the Indian fossil. The plantar margin of the medial hypotarsal crest is parallel to the shaft. *Pelecanus philippensis* has a concave area bounded by a ridge (with pneumatic foramina) on the proximal face of the medial hypotarsal crest. That ridge is more squared off than the subcircular outline in the fossil.

The hypotarsus is not slanted distoplantarly (unlike the state present in the fossil) in the other extant Old World species *Pelecanus onocrotalus*, *P*. *crispus*, and *P*. *rufescens* (fig. 11 in [23]). The tarsometatarsus of *P*. *crispus*, *P*. *onocrotalus*, and *P*. *conspicillatus* are all longer than the Siwalik Group fossil, and the fossil is longer than that of the tarsometatarsus of *P*. *rufescens* (Table 1). The hypotarsus also is mediolaterally narrower in the Siwalik Group specimen than the state in the holotype of the fossil *Pelecanus aethiopicus* [23]. Harrison and Walker [23] illustrate two fully enclosed hypotarsus canals with two additional tendinal grooves on the lateral surface of the medial hypotarsal ridge in *P*. *crispus* and *P*. *rufescens*, and a similar state is present in *P*. *philippensis* (see Figure 3). That state of the canals and grooves appears to be absent in *P*. *onocrotalus* with only one groove on the lateral surface (fig. 11 in [23]). Thus, that combination of hypotarsal canals and its distribution on the topology of the molecular phylogeny [2] may indicate that the two canals and two grooves state is a synapomorphy of the extant Old World species excluding *P*. *onocrotalus*, (i.e. node “E” in ref. [2]).

## Discussion

While two species of extinct pelicans have been known from the Siwaliks for over 100 years, their distinction from and relationships to other species have not been clear. Lydekker [10] emphasized the depth of radial depression as diagnostic, but Harrison and Walker [1] indicated that this character likely represents intraspecific variation. They instead focused on the shape of the ulnar shaft (along with size) as the discriminating features of these extinct species [1]. However, the utility of the previously published Siwalik pelican fossils for diagnostic and comparative purposes is rather limited and also is hindered by the osteological similarity among pelican species as noted by Louchart [8] and Rich and Van Tets [4].

Despite those limitations, the pelican tarsometatarsus is morphologically more variable than other parts of the skeleton and adds significant data that suggests that minimally at least one of the (named) fossil pelican species from the Siwalik Group may be distinct from the extant and extinct taxa from elsewhere in the world (or possibly that it is from another currently unrecognized extinct species). However, only one of the characters discussed above may indicate the phylogenetic relationship of this fossil to known pelicans (Table 2). The absence of the concave face (and its bounding ridge) on the proximal end of the medial hypotarsal crest in *Pelecanus occidentalis* and *P. erythrorhynchos* may be a synapomorphy of the New World pelican clade, but we have not been able to examine specimens of *Pelecanus thagus* to confirm its (likely) presence in that species. Conversely, the presence of that ridge might instead be a synapomorphy of the Old World clade (with its absence as a symplesiomorphy of the New World clade) since it is present in all species of that clade (described above, illustrated by Harrison and Walker [23], and see Figure 3). Given that the polarity of that character is unknown (with no clear outgroup state), which clade is supported by the derived state is uncertain. The same situation may be true of the squared off medio-plantar projection on the proximal end in *Pelecanus erythrorhynchos* and *P*. *occidentalis* (that is absent in other pelican taxa examined; Figure 3), but that trait might vary intraspecifically as some other characters do (e.g., Table 2). Variability in the distal extension of the trochleae, length of the inner ridge of the extensor retinaculum adjacent to the dorsal pneumatic opening, and the ‘slant’ of the medial crest of the hypotarsus all appear to be homoplastic characters among pelicans (Table 2), and useful only in combination for differentiating individual species from each other. Additionally, the combination of two enclosed hypotarsal canals with two tendinal grooves on the lateral surface of the medial hypotarsal ridge may be a derived feature uniting the *Pelecanus sivalensis*? tarsometatarsus with the clade formed by *P*. *crispus*, *P*. *conspicillatus*, *P*. *philippensis*, and *P*. *rufescens* given that state's shared presence among those taxa. Despite that degree of morphological variation, the combination of traits in the new tarsometatarsus from the Siwalik Group indicates that it is distinct from tarsometatarsi of other known pelican species, that it may belong to the Old World clade of crown group pelicans, and that based on its size relative to other specimens, it may represent an individual of *Pelecanus sivalensis*.
